# Sepsis: From Pathophysiology to Individualized Patient Care

**DOI:** 10.1155/2015/510436

**Published:** 2015-07-15

**Authors:** Ildikó László, Domonkos Trásy, Zsolt Molnár, János Fazakas

**Affiliations:** ^1^Department of Anaesthesiology and Intensive Therapy, Faculty of Medicine, University of Szeged, Szeged 6725, Hungary; ^2^Department of Transplantation and Surgery, Faculty of Medicine, Semmelweis University, Budapest 1082, Hungary

## Abstract

Sepsis has become a major health economic issue, with more patients dying in hospitals due to sepsis related complications compared to breast and colorectal cancer together. Despite extensive research in order to improve outcome in sepsis over the last few decades, results of large multicenter studies were by-and-large very disappointing. This fiasco can be explained by several factors, but one of the most important reasons is the uncertain definition of sepsis resulting in very heterogeneous patient populations, and the lack of understanding of pathophysiology, which is mainly based on the imbalance in the host-immune response. However, this heroic research work has not been in vain. Putting the results of positive and negative studies into context, we can now approach sepsis in a different concept, which may lead us to new perspectives in diagnostics and treatment. While decision making based on conventional sepsis definitions can inevitably lead to false judgment due to the heterogeneity of patients, new concepts based on currently gained knowledge in immunology may help to tailor assessment and treatment of these patients to their actual needs. Summarizing where we stand at present and what the future may hold are the purpose of this review.

## 1. Introduction

One of the most challenging tasks in critical care medicine is the treatment of serious infection related multiple organ dysfunction, termed in general as sepsis, severe sepsis, and septic shock. However, sepsis means a very heterogeneous patient population, which varies in etiology and severity; therefore, universally applicable diagnostic criteria and treatment algorhythms are difficult to be defined. This heterogeneity proved to be one of the most difficult hurdles that most prospective randomized trials could not concur; hence, they failed to show either clear survival benefit or positive results of single center studies that were later contradicted by large multicenter trials [1]. Nevertheless, sepsis has become a very important health economic issue all around the world.

Furthermore, treating sepsis is a multidisciplinary task. Early recognition and commencing initial steps of resuscitation are inevitable to give the best possible chance for survival, which has to be started on the primary care level: outside the hospital, in the emergency department or on the wards. In the absence of adequate initial management, providing even the highest level of intensive care would be in vain.

Although the results of prospective randomized clinical trials may be disappointing as far as survival is concerned, it is beyond doubt that we have learned a lot about the pathophysiology of sepsis during performing these studies over the last few decades. Understanding the immunological background of the clinical picture is of utmost importance, which enables the clinician to interpret results of diagnostic tests and rationalize treatment modalities in the most appropriate way. To highlight a few of the current novelties in sepsis pathophysiology and potential new perspectives is the purpose of this review.

## 2. Sepsis Is Not a “Definitive” Disease 

In medical school we were brought up in the world of “definitive diagnoses.” This means that patients come in with a certain complaint, the physician after taking medical history, performing physical examination and diagnostic tests, defines the diagnosis and treat the patient accordingly. In the case of a well-defined disease more-or-less the same or similar diagnostic tests and therapeutic interventions are performed all around the world (such as stroke and myocardial infarction). This holds true for most diseases in classical medicine and surgery. However, defining sepsis is not that simple. The term we call “sepsis syndrome” was conceived in a hotel room in Las Vegas in 1980, during the protocol writing of one of the first prospective randomized trials in sepsis, performed by a group of scientists led by the late Roger Bone [2, 3]. Based on the inclusion criteria of this study a statement paper was later published by the same authors titled “Sepsis Syndrome: A Valid Clinical Entity” [3]. However, these classical signs of the “sepsis syndrome,” such as fever/hypothermia, leukocytosis/leukopenia, tachycardia, and hypotension, meant a very large and nonspecific/noninfectious cohort of patients. For this reason, a few years later a consensus conference was brought together and defined the so called “consensus criteria” of sepsis [4], which has also been recently questioned and criticized by Vincent et al. [5]. In the most current Surviving Sepsis Campaign Guideline a more robust, more detailed definition has been created, in order to “save” the previous concept of the Bone-criteria [6].

These efforts clearly show that finding the appropriate definition of sepsis has been a continuous challenge for more than 30 years. The difficulty in defining sepsis originates from its pathophysiology, to be discussed in Section 4. This has been recognized by international societies and currently an international Task Force has been working on a new, pathophysiology based sepsis definition. Nevertheless, in most specialties the disease itself is easily diagnosed by a laboratory or radiological test. However, in the case of sepsis it is different, which makes not just the diagnosis but the interpretation of the results of clinical trials and also epidemiological data very difficult.

## 3. Epidemiology

According to recent surveys we treat several folds more critically ill patients on the intensive care units (ICU) worldwide these days as compared to the figures from more than 10 years ago [7]. There seems to be an increase in the incidence of sepsis, with mortality rates of 20–50%, and according to recent data from the United States, sepsis is the single most expensive reason for hospitalization at present [8, 9]. However, it is important to note that reported mortality shows considerable variation across the globe. A recent retrospective analysis from Australia and New Zealand showed an increase in the number of critically ill and septic patients over the last 12 years, with a mortality reduction from more than 30% to less than 20% [7]. In the PROCESS trial from the United States mortality was around 20% [10]. According to these data outcome has improved dramatically over the years. However, results from Europe, both retrospective and prospective, indicate greater mortality of 45–55%, which was also accompanied by a 2- to 3-fold longer ICU and hospital stay [11, 12], as compared to that reported by the two previously mentioned studies. This raises the question of whether the care is better in those countries which reported lower mortality rate or is it the patient selection that causes this difference? Although it is difficult to give a definitive answer, referring to our previous chapter, due to the difficulties in defining sepsis, severe sepsis, and septic shock, one cannot exclude that this difference can be the result of the uncertainties in patient selection, and, in those countries reporting higher mortality rates, sicker patients were included in the “septic shock” cohort.

**Figure 1:**
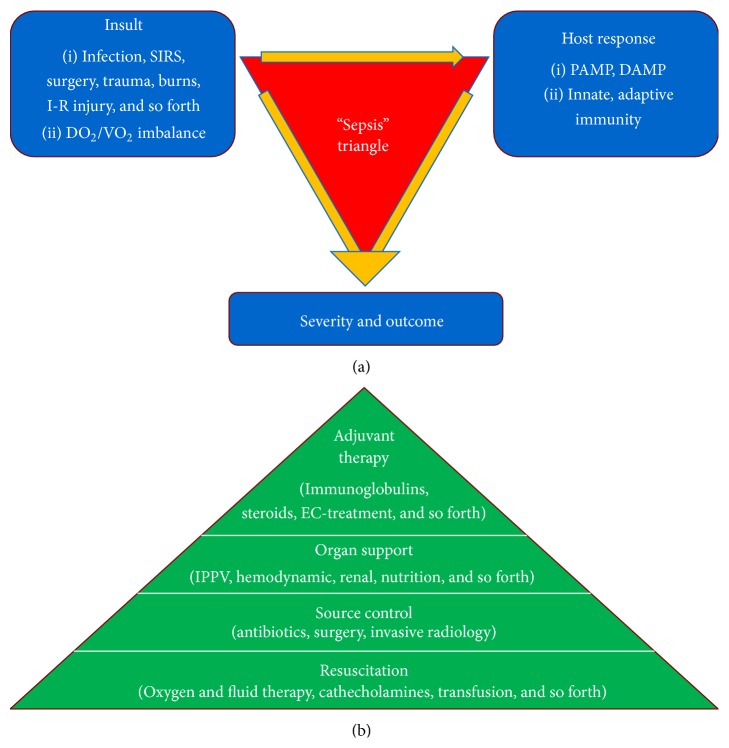
The “sepsis-triangles”: pathomechanism and treatment. SIRS: systemic inflammatory response syndrome, I-R: ischemia-reperfusion, DO_2_: oxygen delivery, VO_2_: oxygen consumption, PAMP: pathogen-associated molecular patterns, DAMP: damage-associated molecular patterns, EC: extra corporeal, and IPPV: intermittent positive pressure ventilation.

Indeed, patients with the same diagnosis of “septic shock” could have completely different severity and prognosis. The same holds true for every potential “insult” in critical care, such as trauma, sterile inflammation (acute pancreatitis), ischemia-reperfusion injury, major surgery, burns, and infection. These conditions share the same feature in their pathophysiology, namely, that it is not the insult* per se*, but the host's response, especially the immune response, which determines severity and outcome (Figure 1).

## 4. Pathophysiology

### 4.1. From Localized Insult to “Cytokine Storm”

The immune system is a “team effort” that involves many different players interacting with each other as an orchestra. The immune response to pathogens relies on both innate and adaptive components. The first line of defense against invaders consists of physical barriers such as the skin [13, 14], the mucous membranes of our gastrointestinal [15], and respiratory [16] and genitourinary [17] tracts. The second line is the rapid defense by the* innate immune system* (including complement proteins, sentinel phagocyte cells, and natural killer cells), which plays an activator and a controller role of the* adaptive immune system* [18]. The innate system acts by broad recognition of antigens, mainly by sensing pathogen-associated molecular patterns (PAMP) of carbohydrates and fatty acids located on the surfaces of common pathogens. By-and-large when a local response spread systemically the activation of several classes of pattern recognition receptors will generate a “cytokine-chemokine storm” [19]. However, very similar molecules are released due to cell injury after trauma, burns, ischemia-reperfusion, pancreatitis, major surgery, and so forth, derived from necrotic cells, mainly from the mitochondria. These are called “damage-associated molecular patterns” (DAMP). It was a very important recognition that after cellular injury similar proteins will be released during bacterial infection, because the genetic background of the bacteria and the mitochondria is very similar [20]. This highlights the fact that the Bone-concept inevitably mixed patients who suffered insults due to PAMP, DAMP, or the mixture of the two.

Activation of neutrophils, macrophages, and monocytes by costimulatory molecules at the site of infection will turn the local adaptive immune system on and give “permission” to the adaptive system to respond to an infectious insult. The aim of the innate response is the eradication of the DAMP and PAMP, which is followed by the adaptive response with the resolution of the immunological process. The adaptive immune response is based on maturation and proliferation, both influenced by the “cytokine signature” of the innate response. In other words, every host has its own “cytokine signature” for a certain insult. Under normal circumstances these processes are well regulated maintaining an even balance between counteracting forces, hence keeping the inflammatory response localized.

**Figure 2:**
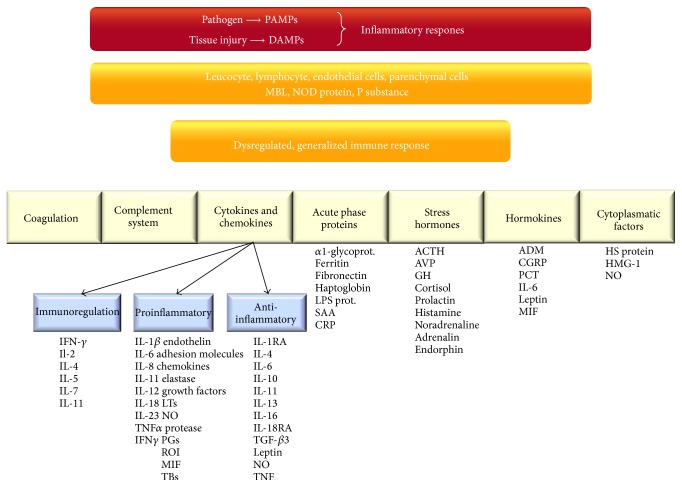
The main pillars of systemic inflammatory response. PAMPs: pathogen-associated molecular pattern, DAMPs: damage-associated molecular pattern molecules, MBL: mannose-binding lectin, NOD protein: nucleotide-binding oligomerization domain protein, and NALP: a type a NOD like receptors. For explanation, see text.

However, in the case of an unbalanced (proinflammatory and anti-inflammatory), dysregulated (maturation and proliferation) response, the localized process goes out of control and becomes systemic, in other words the disease of the whole body; hence, it gives way for impairing the function of distant vital organs. This makes the clinical manifestation of critical illness so similar regardless of the insult. To give an example, the same gravity of acute respiratory distress syndrome (ARDS), shock, or deterioration in mental function can occur in pancreatitis, just as well as after major surgery, or due to any type of infection (Figure 2). The adaptive immune system as the third level of defense is based on its memories. It can adapt and protect us against almost any invader.

In brief the “cytokine signature” of neutrophils and macrophages will give signals to the T and B lymphocytes via the dendritic cells, which after proliferation by maturation will express different cell surface receptors in soluble or membrane bound forms. The adaptive immune response is a soluble matrix, which consists of the cascade-type activation of cytokines, coagulation factors, the release of acute phase proteins, stress hormones, and different chemokines and hormokines, forming a complex network. The key factor of immune resolution is the balance between proinflammatory and anti-inflammatory forces, which is mainly determined by the balance between the relationship of Th1, Th2, Th17, and *γ*ΔT to each other, namely, the maturation, magnitude, and the duration of their activity [19].

### 4.2. Systemic Inflammatory Response Syndrome (SIRS) and Immunoparalysis

Based on the Bone-criteria, systemic inflammatory response syndrome, invented on the Consensus Conference in 1991 [4], initially meant the classical “sepsis syndrome” criteria, without proven infection. The SIRS-criteria have also been criticized for similar reasons as the “sepsis syndrome” definition, but nevertheless this “SIRS-concept” assumed that systemic inflammatory response can occur for an insult without infection.

In the past SIRS was mainly thought to be related to the imbalance between the* proinflammatory *and* anti-inflammatory* responses. However, it is more complex. In the context of the innate and adaptive immune responses both proinflammatory and anti-inflammatory processes take place in a parallel fashion. When the proinflammatory and anti-inflammatory forces swing into action, the proinflammatory forces overwhelm the anti-inflammatory process at the beginning. In general we can say that there is a short delay of the anti-inflammatory response as compared to the proinflammatory. This proinflammatory “dominance” lasts for 2 to 4 days, but an oversized response, which means that the localized insult becomes systemic, will lead to different degree of tissue damage, shock, and eventually organ failure. During the course of disease the adaptive response is initiated by Th1 reaction. In the next phase, the proinflammatory process slowly “turns itself off,” while the adaptive response will switch to a Th2 response. In other words, this later phase helps to survive the proinflammatory process after the eradication of the insult with “*restitutio ad integrum*” [21]. However, a dysregulated, systemic form of the adaptive response could later induce immunoparalysis, jeopardizing the body's defense, hence leaving it prone to further, even opportunistic infections. There are many unanswered questions in this process, but discussing these issues in details goes well beyond the scope of this paper [22].

### 4.3. The Altered Immune Response and Leukocyte Reprogramming

In the later phase, in septic patients and in patients with severe noninfectious SIRS (such as burns, trauma, major surgery, hemorrhage, or ischemia-reperfusion after cardiac arrest), the anti-inflammatory process may overwhelm the proinflammatory forces. This is often referred to as “anergy,” “endotoxin intolerance,” “immunoparalysis,” or “immunodepression,” but these are very general and simplified descriptions of what is really happening. The term* cellular reprogramming* may be more accurate indicating the cellular changes during this process. In brief, cellular reprogramming means two contradictory parallel cellular processes: cells derived from hematopoietic compartments, such as bone marrow, spleen, lymph nodes, and blood, become hyporeactive. In contrast, cells derived from other tissues and solid organs (like liver, kidney, lung, brain, or gastrointestinal tract) can often be hyperreactive causing hyperinflammation in the particular organs, especially in the infected organ. The inhibition of some signaling pathways parallel with others, which are maintained or enhanced, results in large variety of immune response. Immunosuppression itself does not cause harm but leaves the patient prone to infection. Unfortunately, tests able to measure the degree of immunosuppression are not available all around the clock; hence, the clinician has nothing else to rely on at the bedside than the etiology, clinical picture, and biomarkers in order to detect the onset of a potentially devastating new infection as soon as possible [21, 22].

## 5. Diagnostic Challenges

One of the most common misconceptions in sepsis diagnosis is that we have been searching for specific “marker(s) of sepsis.” However, there is not and there will never be one single marker which is able to diagnose sepsis, mainly due to the very colorful manifestation of sepsis and due to the heterogeneity of patients.

Recognizing the septic patient has two main elements. On the one hand, we have to evaluate vital organ function and the degree of organ dysfunction via objective signs, such as hypotension, hypoperfusion, altered mental status, acid-base imbalance, hypoxemia, lactate levels, renal and liver dysfunction, and thrombocytopenia. Based on these findings we should start supportive therapy without any further delay, and if there is any suspicion of the possibility of an infection, empirical antibiotic therapy should also be started immediately (Figure 1) [16].

In the meantime we have to diagnose the etiology of critical illness. In other words we have to decide whether critical illness is* due to infection or not*. Because if it is due to infection antibiotics should be started as soon as possible, but if it is not related to a bacterial infection, antibiotics are not just a waste of time and money, but they may also do harm in short and long term. Unfortunately, diagnosing infection in critically ill patients is not easy.

### 5.1. Conventional Markers of Infection

Clinical signs are the most important in recognizing critical illness, but they cannot prove infection on their own. Conventional (fever/hypothermia, leukocytosis/leukopenia, tachypnoe, tachycardia, and hypotension) indicators, also listed in the classical “sepsis-syndrome” criteria, are very nonspecific, in fact poor indicators of infection. For microbiological proof of infection, although very important, unfortunately results become available 24–48 hours at the earliest after sending the specimen to the laboratory. According to our current concept, it is of utmost importance to start adequate antibiotic therapy as soon as possible, but at least within an hour after the onset of infection caused hypotension; otherwise chances for survival are reducing by the hour [23].

New molecular biology techniques are now available to define the presence of bacterial or fungal DNA within the bloodstream of patients [24, 25]. Highly sophisticated molecular biology based tests such as polymerase chain reaction (PCR), matrix assisted laser desorption/ionization (Maldi/Tof), and peptide nucleic acid fluorescence in situ hybridization (PNAFISH) based pathogen detection can theoretically shorten the recognition of the underlying pathogen to about 8 hours [26]. However, these cannot differentiate between colonization and clinically significant infection. Therefore, we need laboratory tests, which are sensitive and specific enough to show the onset and magnitude of bacterial invasion caused inflammatory response as soon as possible and may also be able to follow the progress of the disease within hours. These biologically active substances are called* biomarkers*.

### 5.2. The Role of Biomarkers at the Bedside

There have been several biomarkers developed so far [1], but neither is suitable for all purposes. Every marker has its own merit and limitations. They inevitably can support decision making but they will never be able to differentiate “sepsis” from “SIRS” with a 100% sensitivity and specificity, mainly due to the problems we discussed earlier in details regarding the problems of defining sepsis, and also due to the complex, overlapping pathomechanism of PAMP and DAMP. Nevertheless, there is still an ongoing search for better, new markers of inflammatory response and infection, with promising preliminary results [40].

There are almost 200 so-called sepsis markers; therefore, discussing the features of those cannot be integrated into the current review. We will mainly focus on the two most commonly used markers: procalcitonin (PCT) and C-reactive protein (CRP). However, briefly mentioning the main features of a few other new markers already applied in daily practice, such as soluble CD14 subtype (presepsin) and soluble urokinase-type plasminogen activator receptor (suPAR), may be worthwhile. Higher presepsin concentrations in septic patients were associated with ICU mortality in a recent large multicenter trial [41]. It was also suggested that changes in plasma concentrations may reflect the appropriateness of antibiotic therapy, but this have to be confirmed by future studies [41]. Regarding the suPAR molecule it has been shown to be a very good indicator of severity of the acute disease and shows good correlation with the degree of organ dysfunction in the critically ill but cannot be regarded as a “sepsis marker” due to its low specificity [42].

Any condition inducing DAMP [43] or PAMP could shed the endothelial glycocalyx layer. It has been confirmed in several experimental studies in different septic models that damage of the endothelial glycocalyx layer is reflected in elevated serum syndecan-1 and syndecan-4 levels [44–47], which may be potentially a very interesting marker in the future, but again, it may be nonspecific for bacterial infection only.

Finally, neutrophil-lymphocyte count ratio is a cheap, fast, and easily available tool to diagnose bacteremia and was found to improve bloodstream infection diagnostics in a recent study on the emergency ward [48]. This simple test may also have a potential in the future.

Nevertheless, the two most commonly used markers in infection/sepsis diagnostics and for guiding therapeutic interventions are PCT and CRP [49]. Despite their popularity, there are still many pros and cons without clear answers regarding their usefulness and interpretation in guiding patient management.

Procalcitonin is detectable in the serum within a few (4–6) hours after the onset of bacterial infection. During the “normal” course of an infection it reaches its peak within 24 hours and then starts its decline in the case of adequate treatment with levels reducing by roughly 50% daily according to its half-life [27]. In contrast, CRP moves “slowly,” and under similar circumstances it reaches its maximum value usually within 48 hours. However, levels are generally elevated in most ICU patients, making interpretation of CRP very difficult [50]. The other major problem with CRP on the ICU is that it is lagging way behind the actual events of the inflammatory process. The most important differences between the two markers are summarized in Table 1.

Procalcitonin differentiates bacterial infections from systemic inflammatory response of other etiologies with higher sensitivity and specificity compared to CRP [39]. There is considerable evidence that PCT supported decision making during antibiotic treatment has several beneficial effects. It considerably reduced antibiotic use in lower respiratory tract infections without compromising survival [51], and it may also shorten the duration of antibiotic treatment on the ICU [52].

Although in the coming paragraphs we will mainly refer to studies investigating PCT, the concept how to interpret these data is potentially applicable for any inflammatory marker and should be taken into account when evaluating biomarker levels at the bedside.

## 6. Interpreting PCT

### 6.1. Sepsis Is Different in Surgical and Medical Patients

Although sepsis is often referred to as a “definitive disease” (see above), in a clinical trial published more than 10 years ago, PCT levels were found to be several folds higher in surgical as compared to medical patients in septic shock despite the similar clinical manifestation and severity of the clinical picture [53]. This indicates different degree of inflammatory response depending on etiology. Indeed, there is increasing evidence that, in the case of massive cell injury, such as in severe trauma [54], after major surgery [55], and any ischemia-reperfusion type injury including cardiogenic shock [37], due to the mechanism of DAMP [56], unspecific elevations of PCT levels can typically be seen even in the absence of a bacterial infection [57, 58]. Theoretically, in surgical patients with sepsis DAMP and PAMP take place at the same time leading to an overwhelming inflammatory response, whilst in medical patients it is primarily the activation of the PAMP, resulting in a less extensive inflammatory response, hence lower biomarker levels [53]. In the study by Clec'h et al., the median PCT value in SIRS in medical* versus* surgical patients was 0.3 (0.1–1.0)* versus* 5.7 (2.7–8.3), and in septic shock: 8.4 (3.6–76.0)* versus* 34.0 (7.1–76.0) ng/ml, respectively.

**Figure 3:**
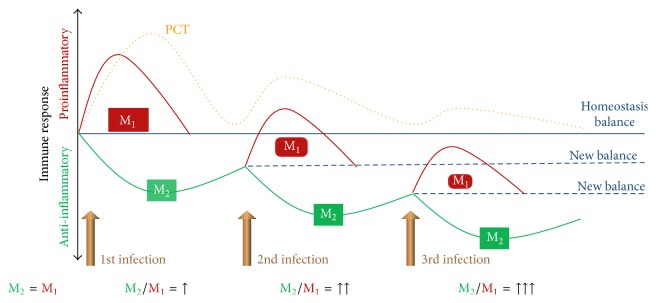
Procalcitonin response to consequent infectious insults. During regulated inflammatory response the two phenotypes of macrophages, (M) the proinflammatory (M_1_) and anti-inflammatory (M_2_), are balanced. As time goes by due to a dysregulated response patients become immunoparalyzed; in other words, M_2_ overwhelms M_1_; hence, forces are shifted towards “new balance.” This is reflected by lower PCT peak levels after each new infectious insult, which can be of the same gravity clinically. For further explanation see text.

Another very important addition to these findings was provided by a study by Charles et al., in which they found different degree of inflammatory response in patients with the first as compared to those with the second septic insult [59]. They investigated patients with primary and secondary blood stream infections and found that the same gravity of infection was accompanied by a severalfold lower maximum PCT level in patients during the second event of infection as compared to those with a primary event. The receiver operating characteristic curve of serum PCT for the diagnosis of blood stream infection in critically ill patients with primary sepsis with a cutoff value of 55.6 ng/mL was 0.934, 95% CI: 0.881–0.970, and in patients with secondary sepsis with a PCT cutoff 6.4 ng/mL it was 0.805, 95% CI: 0.699–0.879. This observation is in accord with what we have already discussed about immunoparalysis and cellular reprogramming in the previous paragraphs put into context with PCT in Figure 3. This shows that lower levels of PCT should be taken seriously in the case of a leter (secon or third) onset of infection and this concept has been further supported by several recent reports [60, 61].

These studies clearly show that a given PCT value should be interpreted differently based on etiology and the time course of the critically ill condition. It is obvious that “one size [of biomarker] does not fit all”; hence, careful evaluation of the given clinical scenario cannot be neglected when interpreting a given laboratory result. Before we discuss the importance of kinetics of biomarkers, let us put the results of recently published clinical trials into this context first.

Recent large clinical trials tested the effectiveness of PCT-guided antibiotic strategies applying the “one size does fit all”; in other words predetermined absolute values (e.g., >1 ng/mL) as a concept and the results were either nonsignificant or patients required mechanical ventilation longer and the prolonged use of antibiotics in the PCT-arm [62, 63]. However, the percentage of surgical patients was around 40% in both studies, and the PCT value indicating the need for an intervention was chosen to be ≥1 ng/mL. Based on the results of previous studies investigating PCT levels in surgical and medical patients, as we discussed before, this 1 ng/mL cutoff value for intervention is a very low PCT value in a specific, mainly high risk surgical population. Indeed, in the study by Layios et al., in patients with a PCT ≥1 ng/mL antibiotics were withheld only in 11%. Although data were not provided for this subgroup of surgical patients, one may assume that these patients received antibiotics unnecessarily in large proportion. The same may hold true for the PASS study that unnecessary antibiotic use, and antibiotic escalation, was inevitable in the PCT-group due to the generally low “alert-PCT” levels (≥1 ng/mL) in the study protocol [63]. However, if a biomarker's half-life is short enough, taking kinetics into account, instead or in addition to their absolute values, may provide several theoretical benefits.

### 6.2. Kinetics over Absolute Values

Although the absolute values of PCT show substantial differences in different etiologies and the course of the disease, but the kinetics may be similar and more useful. Tsangaris et al. studied 50 patients who were in the ICU for more than 10 days, free of infection and who presented with a new onset of fever. Procalcitonin showed a minimum of 2-fold increase in 27 patients from the day before to the day of fever onset, and in these patients infection was eventually proven. On the contrary, infection was not proven in 23 patients in whom PCT remained persistently low and unchanged as compared to previous days. Their conclusion was that a twofold increase of PCT between fever onset and the previous day was associated with proven infection. Furthermore, a normal PCT value on the third day after the fever onset was associated with better survival. It is important to note that the observed maximum PCT values in patients with proven infection remained relatively low (<1.5 ng/mL), but it was not the absolute value but the severalfold increase, which indicated acute onset of infection [64].

This takes us to the importance of PCT kinetics. In most studies PCT kinetics were mainly tested to predict severity and outcome rather than to guide therapy [32, 65]. In a recent pilot study in patients treated on the ICU, we found significant differences in the change of PCT from the day before (Day_−1_) to the day when new infection was suspected according to the clinical picture (Day_0_). On Day_−1_ PCT levels were similar in patients in whom infection was eventually proven as compared to patients in whom we could not prove infection. Although on Day_0_ absolute values of PCT levels were elevated in both groups, levels were significantly higher in patients in whom infection was later proven. Most importantly, while there was no significant change in the levels of PCT from Day_−1_ to Day_0_ in the noninfectious group, the rate of increase was significant in the infection group [34]. It has also been shown that PCT kinetics (>80% drop from its maximum value) can be very useful in stopping antibiotic therapy early, hence reducing antibiotic consumption and length of treatment significantly, which is also recommended in the recent Surviving Sepsis Guideline [6, 52]. These results suggest that therapy based on PCT kinetics may be superior as compared to predefined absolute values, a hypothesis to be tested in the future.

### 6.3. Fungal and Viral Infections

Recent studies show that fungal infections have an increasing tendency in critically ill patients [66, 67]. Candida spp. are the third or fourth most commonly isolated microorganism in the bloodstream of ICU patients and its associated mortality is reported to be as high as 40–60% [67, 68]. Candidemia or invasive candidiasis is defined by positive blood cultures and presence of clinical signs of systemic infection. Fungal infections are difficult to diagnose from blood cultures because it takes a considerable amount of time to grow these organisms and it often remains negative [69, 70]. Unfortunately, clinical features are very nonspecific to separate bacteria-related sepsis from Candida sepsis. A number of clinical trials have proposed the potential diagnostic value of PCT in this context. Martini et al. investigated PCT levels for the diagnosis of candidemia or bacteremia in septic patients. They have found that a low PCT value (0.71 [0.5–1.1] ng/mL *p* = 0.001) in a critically ill septic patient is more likely to be related to candidemia than to bacteremia [71]. Another trial by Cortegiani et al. reported that PCT could be a useful diagnostic tool to separate Candida spp. blood stream infection (0.99 ng/mL, 0.86–1.34) from blood stream infection caused by bacteria (16.7 ng/mL, 7.65–50.2) or in mixed infections (4.76 ng/mL, 2.98–6.08). There was no difference in PCT levels to exclude the detection of Candida spp., by blood culture (alive Candida) and real-time PCR (killed Candida) in septic patients. In this study significantly lower values of PCT were observed in [72].

Marková et al. determined the role of PCT testing in patients with high risk for invasive fungal infection. They included immunocompromised hematological patients undergoing chemotherapy or allogeneic hematopoietic stem cell transplantation and had bacterial or fungal infectious complications. C-reactive protein and PCT were prospectively assessed from the day following fever onset for four consecutive days. They found increased CRP combined with mildly or not elevated PCT in immunocompromised patients probably due to fungal infection. Therefore, the complementary use of these biomarkers may help the diagnostic method [35].

A recent systemic review and meta-analysis summarized current evidence on the role of PCT in differentiating fungal infections from other infectious diseases. They identified 8 eligible studies and summarized 474 episodes of suspected fungal infections. The most frequently isolated causative organisms were* Candida albicans*,* Candida parapsilosis*,* Candida glabrata*,* Candida tropicalis*,* Aspergillus *spp.,* and Penicillium zygomycota*. They found that PCT has a good diagnostic power to separate invasive fungal and bacterial infection from noninfectious disease conditions [73].

Another clinical trial investigated the differences between Gram-negative (G−), Gram-positive (G+), and fungal bloodstream infections. They observed significantly higher PCT levels in patients with G− as compared to G+ infections and even lower levels in fungemia [74].

Regarding viral infections, most of the published results agree that PCT can differentiate between viral and bacterial infections, as levels will remain low in the latter case [75–77]. However, it is important to acknowledge that any condition, including sever viral infection, nonbacterial systemic inflammatory condition such as sterile acute pancreatitis or any ischemia-reperfusion injury, which is accompanied with significant hypotension/hypoperfusion of the tissues, can cause a DAMP-induced PCT increase, which may complicate differential diagnosis [56, 78, 79].

## 7. Treatment: What the Future Holds?

### 7.1. Extracorporeal Removal of Mediators and Toxins

Extracorporeal clearance of the plasma, via hemofiltration and plasma pheresis, has received major interest over the last decades in sepsis research. As the results were contradictory, nowadays the focus of interest has turned towards new alternatives, such as the targeted removal of toxins and mediators via specific adsorption.

#### 7.1.1. Polymyxin-B

Polymyxin-B (PMX-B) is a cyclic cationic polypeptide antibiotic originated from* Bacillus polymyxa*. This antibiotic has the facility to bind and neutralize endotoxins [80]. Studies have shown that PMX-B blunts the TNF-*α* response to endotoxin [81], which is due to the high binding affinity of PMX–B for the LPS molecules. Unfortunately, PMX-B infusion causes nephrotoxicity and neurotoxicity in humans [82]. However, when polymyxin B is linked covalently to a polystyrene-derived fiber in a hemoperfusion cartridge, it can be used to remove circulating endotoxins without exerting its undesired effects systematically. The surface area of the column is extremely large, so it can clear up a large amount of circulating endotoxins in a relatively short period of time [83]. Another potentially beneficial effect of PMX-B hemoperfusion is the removal of certain inflammatory cells [84]. This device has been used and tested in many patients with a very low incidence of adverse events (<1%), such as thrombocytopenia, allergic reactions, and transient hypotension [82]. As PMX-B hemofiltration has been available for several years, it cannot be regarded as a “new” treatment alternative* per se*; nevertheless, it is far from routine use in the everyday practice; hence, future studies are warranted.

#### 7.1.2. CytoSorb

CytoSorb is a hemadsorption device. It removes both proinflammatory and anti-inflammatory cytokines. The cartridge contains biocompatible, greatly porous polymer beads capable of absorbing molecules in the ~10–50 kDa range [85–87].

Cytokine overproduction is a common feature in many life-threatening conditions in addition to sepsis, such as trauma, major surgery in high risk patients, viral infections, acute respiratory distress syndrome (ARDS), serious burn injury, and acute pancreatitis, liver failure just to name a few. Several case reports have been published about the use of CytoSorb treatment over the last couple of years. These include *β*-hemolytic streptococcus-induced necrotizing fasciitis [88], septic shock with multiorgan dysfunction [89], and rhabdomyolysis [90]. Elevated cytokine levels have been reported during donor conditioning for organ transplantation, which were associated with dysfunction of donor organs before and after transplantation [91, 92]. In a recent clinical trial it was found that, in addition to conventional treatment, attenuating the inflammatory response by cytokine absorption, graft survival can be prolonged [91].

### 7.2. Anti-PD-1 Immunotherapy in Sepsis and Tumor Diseases

The late phase of sepsis and the late phase of cancer by-and-large share similar immune suppression mechanism. One of the similarities is based on the presence of negative costimulatory molecules, such as PD-1 (programmed cell death-1). Its expression is induced primarily on T cells' CD4 and CD8 surface proteins, the signaling via which PD-1 inhibits T cell proliferation, cytokine production, and cytotoxic ability. Persistent antigen exposure (DAMP-PAMP) causes increased levels of PD-1 consequently T-cell depletion [93, 94]. Theoretically, blocking the PD-1 receptor or its ligand by antibodies could reverse T cell dysfunction and inhibit the pathogen or tumor cells initiated immunosuppression [94]. Inhibition of the PD-1 pathway in animal models resulted in clinically significant survival benefit in bacterial and fungal sepsis [95, 96]. In a recent clinical trial, patients with lung cancer, melanoma, and small-cell renal cancer patients responded to anti-PD-1 antibody treatment in 20 to 25% [97]. Based on the similar immune-pathomechanism of cancer and sepsis, testing the effect of anti-PD-1 or anti-PD-L1 in the future certainly makes sense. Furthermore, since septic patients do not require long-term anti-PD-1 or anti-PD-L1 therapy the potential adverse effects of certain autoimmune reactions or other serious complications should be very rare. Therefore, future studies are warranted to confirm safety and efficacy issues of anti-PD-1, anti-PD-1L treatment in immunoparalysed septic patients [98]; furthermore, evaluating PD-1 or PD-L1 expression in immune cells may be a useful biomarker for immunomodulatory therapy.

### 7.3. Stem Cells and Genetic Treatment

Bone marrow-derived multipotent mesenchymal stem cells (MSCs) are already in the clinical use in multiple clinical disorders including myocardial infarction [99], diabetes [100], hematological malignancies [101], hepatic [102], and renal failure [103]. Recent animal experiments suggested that bone marrow-derived MSCs may also have a potential role in the treatment of acute renal failure, ARDS, and sepsis [104–106].

In several recent animal models in mice, investigating drug- and ischemia-reperfusion-induced acute kidney injury, MCSs therapy was found to enhance recovery and prolong survival [104, 107, 108]. In other animal models circulating MSCs were able to help to regenerate new renal tubular cells in acute kidney injury [109, 110].

MSCs can also be potentially used in ARDS by attenuating proinflammatory response by regulating both the innate and adaptive immune systems and modulation of macrophages [111]. They can influence activated CD4 and CD8 T cells via the inhibition of the inflammatory cytokine production and stimulate the regulatory T cells. MSCs can directly affect sepsis, one of the most common causes of ARDS, by enhancing macrophage phagocytosis and increasing antimicrobial peptide secretion, thereby increasing bacterial clearance [106, 111]. Another animal experiment showed that MSCs can also help to repair the injured lung following ventilation-induced lung injury [112]. There is increasing evidence about the potential mechanisms via which MSCs act in the injured lung [113]. There is one ongoing multicenter clinical trial on the effects of allogeneic MSCs therapy in patients with moderate to severe ARDS, in which patient recruitment has already started [ClinicalTrials.gov, NCT01775774].

As patients respond differently for seemingly same infectious insults, genetic variants are likely to explain the differential susceptibility in the risk of severe sepsis. It is obvious that host genetics can influence sepsis outcomes but no specific loci have yet been confirmed. This year, the first genome-wide study reported significant correlation between certain single nucleotide polymorphisms and 28-day mortality in intensive care patients with sepsis, severe sepsis, or septic shock [114]. After the exact clarification of some responsible loci and its role in the background, mechanism, and course of sepsis, genetic manipulation may be another potential therapeutic approach of sepsis therapy in the future.

## 8. Conclusion

Understanding the underlying pathology in sepsis and critical illness in general is inevitable in order to evaluate clinical signs and biomarkers in the right context at the bedside. In-depth analysis of recent research shed light on several important issues including the immunological background of host response for different insults summarized in the DAMP and PAMP concept, which also explains why biomarker levels should be interpreted differently based on etiology and why their kinetics may carry more appropriate information than the absolute values. Furthermore, this understanding may lead us to a completely different strategy in treatment where the major role will be played by adsorption techniques and cellular reprograming.

However, this knowledge also revealed that in the complex condition of sepsis nothing will ever replace the well trained, experienced, thinking physician, who takes all of the available information into consideration at the bedside and then makes a decision. Finally, even if this decision will be proved to be wrong retrospectively, it should not be interpreted as a failure, but rather as an important source of our experience. This experience, which contradicted our expectations and disappointed us at the time, leads to the design of several research projects and more importantly it already helped us to understand more about sepsis and changed the way we thought about it 30 years ago, completely.

## Figures and Tables

**Table 1:** Comparison of CRP versus PCT (advantages and disadvantages).

	CRP	PCT
Differentiating bacterial infection from SIRS	− [27]	Specific for bacteria [28, 29]
Response to infection	Slower (days) [27]	2–6 hours [30]
Peak response after infection	2-3 days [27]	12–48 hours [27]
Half-life	Several days [27]	20–35 hours [31]
Plasma kinetic	Slow [27]	Rapid [27]
Price	+	++++
Correlating disease severity and progression	Slightly [27]	+++ [32]
Correlating effective therapy	+	+++ [33, 34]
Prognostic factor for mortality	Weak or nonexistent [27]	Good predictor [31, 32]
Differentiating G+ from G−	− [35]	++ [35]
Response to other factors	Virus, autoimmune diseases, local infections, surgery, trauma [27]	Surgery, trauma, burn, cardiogenic shock, liver cirrhosis [36–38]
Fungal infection	same as bacterial [35]	Slightly elevated [35]
Immunosuppression	Formation can be changed [27]	The induction is reduced [27]
Biological effect	Opsonin for phagocytosis [27]	Chemokine [27]
Sensitivity/specificity	Sensitive but nonspecific [27]	Sensitive and specific [27, 39]
General use	Outpatient care [27]	In intensive care [27]
